# Something about Oxaluria

**Published:** 1882-09

**Authors:** Charles G. Stockton


### SOMETHING ABOUT OXALURIA.*

BY CHARLES G. STOCKTON, M. D.

In the year 1831, Dr. Wm. Prout described the oxalic acid diathesis; and in his lectures and writings announced certain peculiar views on the pathology of assimilation and secretion, which, at that time, attracted much attention.

Prout † held that the presence of oxalic acid in the urine was usually owing to a distinct diathesis, marked by symptoms of the irritable or nervous class rather than to the imflammatory and congestive, attended with irregularity of the heart's action, palpitation, flatulent disorder of the stomach and depression of the spirits reaching sometimes to hypochondriasis; that the primary assimilating processes were largely concerned, and that it was particularly in the mal-assimilation of saccharine aliments, and, perhaps, in extreme cases, those of an albuminous and oleaginous nature that the trouble began.

He stated that this diathesis was present with two classes; individuals of the sanguine temperament on the one hand and those of the melancholic on the other; that when the acid is produced *in the system*, the urine is transparent, pale, greenish yellow in color, free from sediment, and of moderate specific gravity.

Dr. Prout thought that the plan of diet in these cases should be similar to that adopted in diabetes, advising animal and sometimes farinaceous substances, but insisting that sugar should be excluded, and that the eating of it was almost certain to be followed by a crop of oxalates in the urine.

Golding-Bird confirmed and emphasized the views expressed by Prout, invented the term oxaluria, and by his book on urinary and stomach diseases, widely extended his belief.

But there were contemporaries of these men who opposed the theory of the formation of oxalic acid within the economy, accounting for the phenomenon upon the grounds held by Dr.

---

* Read before the Buffalo Medical Club, June 21st, 1882.
† Prout on stomach and urinary diseases, p. 184.

Owen Rees, as stated by him in the "Croonian Lectures" and his book "On the Analysis of the Blood and Urine in Health and Disease," viz., that oxalic acid depended for its origin upon the excessive formation of lithic acid, that the oxalate of lime was formed after the urine was secreted by the kidneys and was derived from the oxidation of uric acid and urates therein.

From the fact that a deposit of oxalate of lime is frequently found in normal urine after a day's exposure to the atmosphere, and, further, from the formation of oxalic acid by mixing yeast with uric acid and exciting fermentation (Wohler) and from somewhat similar experiments by Leibig, Lehman and others—it was, and still is held by many, that the formation of this acid is not within the fluids of the body at all, but in a decomposition of uric acid after the secretion of the urine, only.

Dr. Basham* presents this side of the argument very ably, saying: "The symptoms, then, of the disorder which Golding-Bird designated as oxaluria are closely associated with, if not dependent upon, a state of the organism which Dr. Murchison has very appropriately termed *lithæmia*, a condition in which oxidation is imperfectly performed and insoluble combinations of lithic acid and lithates, instead of readily soluable urea results," and further on he explains that this condition produces all the symptoms, frequent micturition, palpitation of the heart &c., described by Prout in his diathesis.

It would appear to a man having some experience with oxaluria that the position taken by Dr. Basham, *et al*, while possessing much truth, can be proven in error both in theory and practice, that, in point of fact, both Prout and Owen Rees are partly correct, that the formation of oxalic acid may be from abnormal metamorphosis occurring in the blood as stated by one, and also from changes taking place in the urine after secretion, but before excretion as claimed by the other.

And again, it would appear that the typical representative of the first class is a melancholic, nervous individual having cold

* Reynold's System of Medicine, Vol. III.

hands and feet, flushed and aching head, coated tongue, weak and rebellious stomach and heart; having rather too free secretion and frequent discharges of pale, greenish colored, transparent urine, acid in its reaction; having occasional attacks of renal colic and perhaps the passage of a calculus of calcium oxalate.

Eating saccharine substances and drinking hard water always exaggerate his sufferings.

The typical representative of the second class, once a sanguine, robust, active and stomach-loving man, adopts sedentary habits of life, following which he has sour stomach, constipated bowels, a peevish disposition, urine of deep color, high specific gravity, and loaded with uric acid, oxalate of lime and urates—frequent attacks of nephritic colic and discharges of gravel, which concretions are mixed, uric acid and oxalate of lime. This man has all the signs of lithæmia and finds greatest comfort by conforming to a non-nitrogenous diet.

Such illustrative cases are extreme, and all agree that these conditions may be satisfied with typical completeness without the presence of oxaluria; and, conversely, one may have oxaluria and even the formation of an oxalate of lime calculus with the development of none of these symptoms.

Dr. Wm. Roberts * says: I am strongly convinced that oxaluria arises from a variety of conditions, many of them not accompanied by appreciable departures from health, in which the assimilation of food or the disintegration of tissue goes on imperfectly; and that it is impossible to assign any constant train of symptoms as the cause or the consequence of oxaluria."

From these facts and considerations we may reach the following conclusions, which seem substantiated.

First.—Oxalic acid may be present in the blood as a result of mal-assimilation or imperfect disintegration; it may be eliminated by the kidneys in the form of oxalate of lime, thus producing oxaluria.

---

* Roberts Urinary and Renal Diseases, p. 81,-1876.

Second.—Oxalic acid may be formed from the decomposition of uric acid after its secretion by the kidneys and appear in the urine in the form of oxalate of lime.

To prove the first proposition there are many authorities to draw upon, besides some personal experience.

In a case, wherein, as a result of malaria and improper diet, there gradually appeared signs of indigestion with palpitation, head-ache, and frequent micturition, the urine became abundant pale, acrid and loaded with oxalates. These features were found to be aggravated by eating sugar or taking carbonated drinks, and to be alleviated when large quantities of pure water were taken, and the diet limited to a azotized or farinaceous foods.

The following articles are named in order with relation to their noxious effects:—Fermented cider, maple syrup or honey, cane sugar, soda water with syrups, lager beer and other carbonated drinks. Fat and lean meats and starchy foods (especially when taken with extract of malt) were assimilated with comfort.

Now in this case there was not, at any time, marked excess of uric acid in the urine, and probably not in the blood, while there was marked oxaluria, the discharge of a pure oxalate of lime calculus and probably oxalic acid in the blood. Notwithstanding the statements of Dr. Owen Rees that oxalic acid is not found in the blood, we know to the contrary, because, after oxalic acid is taken into the stomach, it rapidly appears in the urine; and moreover, because Dr. Garrod has repeatedly detected this acid in the fresh blood and sweat. This observer,* in 1876, writes as follows: "Some years since I detected oxalic acid in the blood of a patient laboring under albuminuria. * * * The acid was separated from the watery solution of the serum, mostly in the form of octohedral crystals of oxalate of lime, but mixed with small oval bodies, somewhat resembling the dumb-bell varieties of this salt. I have also examined the blood for this acid in many cases of gout and have frequently detected it; my impression is that it chiefly occurs during the inflammatory

* Garrod on Gout and Rheumatic Gout, p. 112, stage of the disease, and is probably derived from the oxidation of the uric acid. The whole subject, however, is beset with difficulties, and, until many observations have been made, I should not wish to venture any positive statement, except as to the fact of the frequent occurrence of oxalic acid in the blood." The experiments of this writer regarding oxalic acid in the sweat are worthy of perusal and may be found in his work referred to above. Several cases have been reported in which oxalate of lime was found within the substance of the kidney.*

Oxalic acid is found in the urine after the injection of urates into the veins.†

Probably enough is already said to verify the first of our conclusions, but further evidence is readily accumulated from the works of English and Continental writers.

Our second proposition seems to be admitted by all recent writers; and the fact that oxalic acid frequently does appear, cannot be a matter of surprise when it is remembered that oxalic acid results from the oxidation of uric acid.

This abstract from Liebig‡ is worth reading. "It is a common occurrence in France among patients suffering from calculous complaints, that, when they go to the country, where they take more exercise, compounds of uric acid, which were deposited in the bladder during their residence in town, are succeeded by oxalates in consequence of the increased supply of oxygen. With a still greater supply of oxygen they would have yielded in healthy subjects only the last product of the oxidation of uric acid, namely, carbonic acid and urea."

Whether or not oxalic acid appears in the blood as the result of the oxidation of uric acid, it unquestionably exists as the result of such oxidation after the secretion of the urine, for it is a common observation that normal urine, when exposed for a few hours to the atmosphere, shows an abundance of oxalate of lime crystals at the expense of uric acid.

* Ziemssen's Encyclopedia, vol. xv, p. 690.

† Draper's Physiology, p. 222.

‡ Uric Acid and Urea, p. 45.

Thus oxalic acid, as it appears in the urine, represents an intermediate stage in the oxidation of the more complex organic substances; and whenever oxidation is seriously retarded, we may expect its presence, and besides in indigestion, it often appears in anæmia, the convalescence of severe fevers and chronic pulmonary diseases, also, after drinking certain carbonated beverages, as champagne, cider, etc.

I can find no authority to that effect, but I strongly suspect that quinine aggravates the disorder when taken during an attack of oxaluria, even if it does not sometimes bring about the same; which suspicion will not appear unnatural when we reflect that, according to accepted investigators, quinine lowers the temperature by lessening the oxidizing function of the blood.

Agents that assist or retard oxidation might be discussed with advantage, as also might the treatment of, and the proper diet to be observed in cases of oxaluria, but these matters are omitted as I have already, in accordance with the title of the paper, said "Somthing about Oxaluria."

